# Correlation Between Corneal Whorl-Like Nerve and Retinal Neurodegenerative Changes and Their Association With Microvessel Perfusion in Diabetes

**DOI:** 10.1167/iovs.64.14.44

**Published:** 2023-11-29

**Authors:** Dongyu Li, Xin Jin, Chao Wang, Nan Zhang, Di Jin, Hong Zhang

**Affiliations:** 1 Eye Hospital, The First Affiliated Hospital of Harbin Medical University, Harbin City, Nangang District, Heilongjiang Province, China

**Keywords:** cornea, diabetic retinopathy (DR), in vivo confocal microscopy, diabetic peripheral neuropathy, optical coherence tomography angiography (OCTA), retinal neurodegeneration

## Abstract

**Purpose:**

The purpose of this study was to compare the evolution of changes in the corneal nerves, retinal nerves, and cells and blood vessels at a single time point in early diabetic retinopathy (DR).

**Methods:**

Eighty participants (60 with diabetes and 20 nondiabetic controls) were examined. DR was graded according to the International Classification of Diabetic Retinopathy. Inferior whorl length (IWL), spiral orientation, central nerve fiber length (CNFL), retinal nerve fiber layer (RNFL) thickness, ganglion cell complex (GCC) layer thickness, global loss volume (GLV), focal loss volume (FLV) indices, superficial (sVD), and deep vessel densities (dVD) were examined.

**Results:**

Compared with those of healthy controls, the IWL, CNFL, and FLV were decreased in the diabetic groups (*P* < 0.001). The IWL was significantly positively correlated with the RNFL and GCC thicknesses in the diabetic group (*r* = 0.248, *P* = 0.006 and *r* = 0.207, *P* = 0.023, respectively) and significantly negatively correlated with the FLV (*r* = −0.535, *P* < 0.001). The sVD was significantly positively correlated with the RNFL thickness (*r* = 0.314, *P* < 0.001) and negatively correlated with the GLV (*r* = −0.229, *P* = 0.012).

**Conclusions:**

Our findings suggest a correlation between corneal whorl-like nerve plexus and retinal nerve changes in the early stages of DR and that the IWL of the cornea may be able to indicate the extent of DR. Retinal nerve changes are associated with retinal microvessel perfusion, and nerve changes may precede vessel lesions.

Traditionally, diabetic retinopathy (DR) has been defined as a microvessel complication of diabetes mellitus and is usually diagnosed clinically based on fundoscopic findings of diabetic microaneurysms. However, there is increasing evidence that damage to the ocular nerve occurs before the onset of clinical symptoms in patients with diabetes^1^ and that neurodegenerative changes play an important role in the pathophysiology of DR; some studies have even suggested that neurodegenerative changes can occur before the onset of vessel disease.^2^ Optical coherence tomography angiography (OCTA) can accurately identify and detect retinal neurodegenerative and microcirculatory changes by quantifying structural changes and vessel density in specific retinal layers.^3^^–^^6^

In addition to those of the retinal nerves, degenerative changes caused by diabetes also occur in the corneal nerves, and, as the cornea is one of the most densely distributed structures of nerves throughout the body, previous studies have demonstrated corneal nerves can be quantified by in vivo confocal microscopy (IVCM), providing a diagnostic and prognostic reference for patients with diabetes.^7^ Studies have demonstrated that the corneal nerve fiber length (CNFL) and corneal nerve fiber density (CNFD) can be objectively, accurately, and reliably used to assess the degree of diabetic peripheral neuropathy (DPN) by scanning the basal nerve plexus around the central cornea.^8^^,^^9^ In contrast, other recent studies have shown that the severity of nerve damage is higher in the cornea inferior whorl length (IWL) than in the central region.^10^ The inferior whorl is a region of swirling structures located slightly inferior to the corneal apex to the nasal side. In the assessment of neuropathy, its utility is comparable to or even higher than that of the central zone corneal nerve fiber parameters.^11^ Because of its unique appearance, some scholars believe that the length of the inferior whorl may be a more reliable marker for the evaluation of the corneal sub-basal plexus.^12^^–^^15^

Therefore, the purpose of this study was to compare the evolution of changes in the corneal nerves, retinal nerves, and cells and blood vessels at a single time point in early DR, with the aim of diagnosing DR early and indicating the extent of DR lesions by noninvasive examination of the cornea.

## Materials and Methods

The study was approved by the Ethics Committee of the First Affiliated Hospital of Harbin Medical University (IRB-AF/SC-04/02.0) and was conducted according to the principles of the Declaration of Helsinki as revised in 2008. People with type 2 diabetes were recruited from the First Affiliated Hospital of Harbin Medical University, and 80 subjects (including 20 gender- and age-matched volunteers without other medical conditions and 60 patients with diabetes) were examined. The type of diabetes was determined from the endocrinologist's report, and information on the course of diabetes was derived from the patient's self-description.

In this cross-sectional study, we included adult patients with confirmed type 2 diabetes and no signs of DR fundoscopy and patients with mild, moderate, or severe nonproliferative diabetic retinopathy (NPDR) obtained according to the International Staging Criteria for Diabetic Retinopathy.^16^ Diabetes but no DR was defined as no abnormalities. Mild NPDR was defined as microaneurysms only. More than just microaneurysms but less than severe NPDR was defined as moderate NPDR. Besides, any of the following: more than 20 intraretinal hemorrhages in each of 4 quadrants; definite venous beading in 2 quadrants; Prominent intraretinal microvascular abnormalities in 1 quadrant, and no signs of proliferative retinopathy was defined as severe NPDR. Nondiabetic participants were considered as the control group. There are 20 participants that were identified as having diabetes but no DR, 20 participants had mild to moderate NPDR, and 20 participants had severe NPDR. The patients with proliferative DR were not included because this study was designed to study patients with early-stage DR, and severe NPDR is a vision-threatening type with a poor prognosis, severe NPDR was grouped separately.

Exclusion criteria included: (1) patients with proliferative DR, (2) refractive media clouding, (3) structural damage to the macular center, (4) active intraocular inflammation, (5) any other history of neurological disease and any systemic disease other than diabetes affecting the cornea, and (6) patients with ocular trauma, history of surgery, and other ocular diseases.

### In Vivo Confocal Microscopy

**Figure 1.**
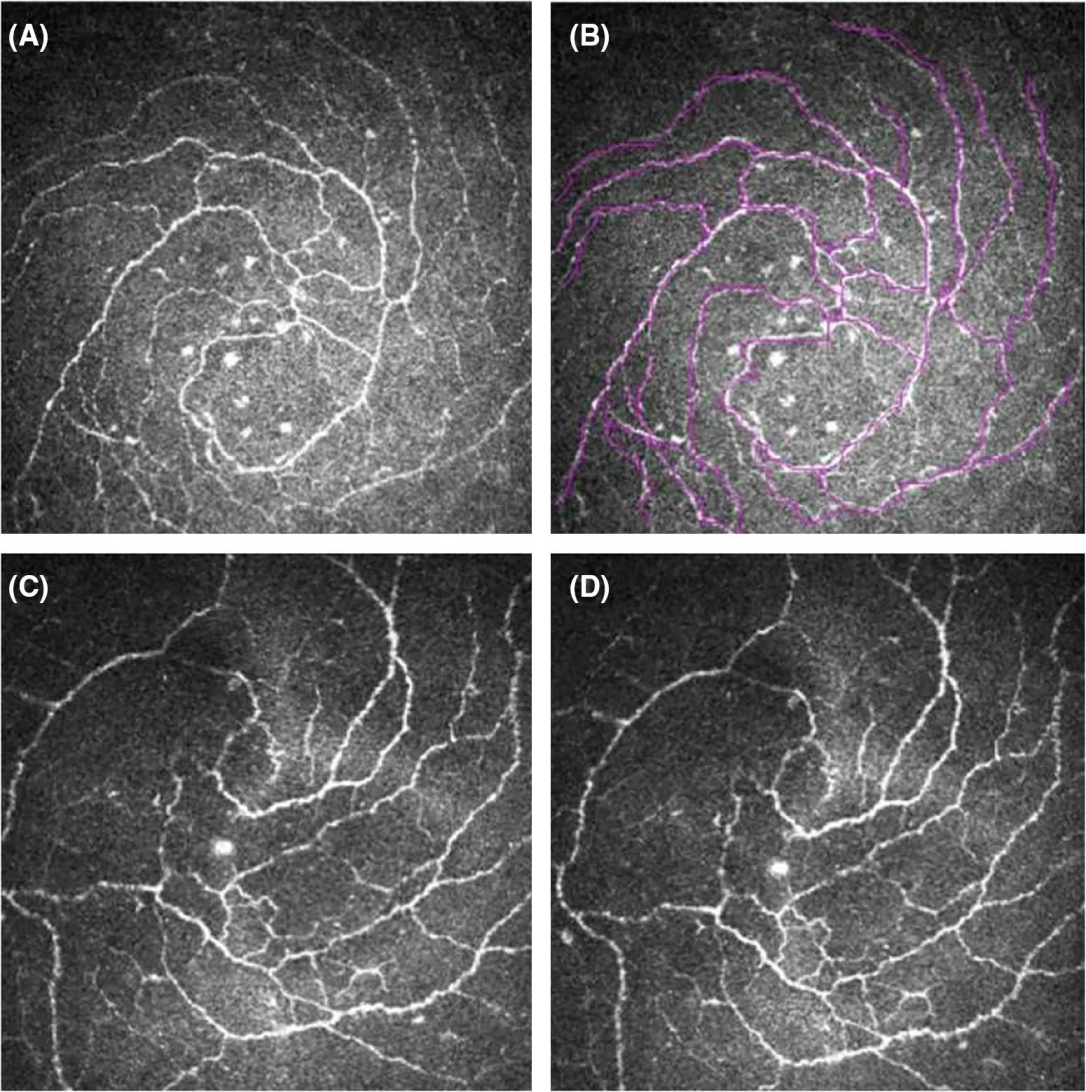
**In vivo confocal microscopy images of nerves in the corneal whorl-like area.** Note: (**A**) Original image. (**B**) Images after applying Neuron-j software processing. (**C**) The image chosen by one experimenter to represent the center of the whorl-like area. (**D**) The image chosen by another experimenter to represent the center of the whorl-like area.

Laser scanning IVCM (Heidelberg Retina Tomograph 3 with Rostock Cornea Module, Heidelberg Engineering GmbH, Heidelberg, Germany) images of the whorl-like area of cornea were obtained from all subjects. This microscope uses a 670-nm wavelength diode laser source, and it is equipped with a 63 × objective immersion lens with a numerical aperture of 0.9, allowing a scanning area of 400 × 400 mm with a magnification of up to 800 times and a resolution of approximately 1 mm. Before the examination, one drop of 0.4% oxybuprocaine hydrochloride (Benoxil; Santen Pharmaceutical, Japan) was applied to the lower conjunctival sac. With the nerves of the sub-basal plexus visible in the real-time display of the microscope, images of the plexus were automatically recorded at 3 to 8 frames/second as the operator adjusted the field of view to locate the inferior whorl region. Fine depth adjustments were made to maximize the visibility of the whorl region during acquisition, to enable later analysis of the predominant orientation of the sub-basal nerves. A semi-automatic tracer program Neuron J (a plug-in for ImageJ; Erik Meijering, Rotterdam, The Netherlands) was used, which is public domain image analysis software distributed by the National Institutes of Health (Bethesda, MD, USA). The IVCM parameter was the CNFL and IWL of the cornea nerve plexus. To assess interobserver agreement, two observers (masked to each other) were asked to determine from their set of captured images which image best represents the whorl center and then calculate the IWL independently. The IWL is calculated by adding the lengths of all corneal nerve fibers and branches captured in the image and dividing by the one image's squared measurements (Fig. 1).

### Retinopathy Assessment

OCTA utilized the RTVue imaging device (Optovue, Inc., Fremont, CA, USA) to perform ONH mode which acquires a 3.45 mm diameter circle scan along the optic nerve head for the surrounding retinal nerve fiber layer (RNFL) thickness measurement. Microcirculation was assessed by analyzing the vessel density of the 6.0 × 6.0 mm HD scans and calculated as the percentage of area occupied by vessels in the selected area, and superficial (sVD) and deep vessel density (dVD) were assessed in the macular region.

The ganglion cell complex (GCC) pattern measures the thickness of the GCC at the macula, which is a composite of the inner plexiform layer, ganglion cell layer, and nerve fiber layer. This system displays individual GCC thickness, deviation, and significance maps for GCC measurements. In addition, the study evaluated the GCC parameters based on the model, namely the focal loss volume (GCC FLV) and the global loss volume (GCC GLV). One of the parameters of GCC, FLV, is the mean value calculated by comparing with the age-matched normative database and reflects the local loss in the GCC map of both eyes of the subject. GLV, on the other hand, is the mean value derived from calculating the loss in the whole GCC map, reflecting the overall loss in the GCC map of the subject's eyes.^17^

### Statistics

SPSS version 23.0 was used for statistical analysis in this study. The measures obeyed normal distribution and were expressed as mean ± standard deviation, 1-way ANOVA was used for comparison among the three groups, and the LSD-*t* test was used for multiple comparisons between two groups. FLV, GLV, IWL, CNFL, and GCC were non-normally distributed and expressed as median (lower quartile and upper quartile; M [P25 and P75]). Comparisons among the three groups were made using the Kruskal-Wallis H test, and multiple comparisons between the two groups were corrected using the Bonferroni (post hoc test) method. The cardinality test was used for the comparison of the count data. Spearman's rank correlation analysis was used for correlation analysis among factors, and *P* < 0.05 was considered a statistically significant difference.

## Results

**Figure 2.**
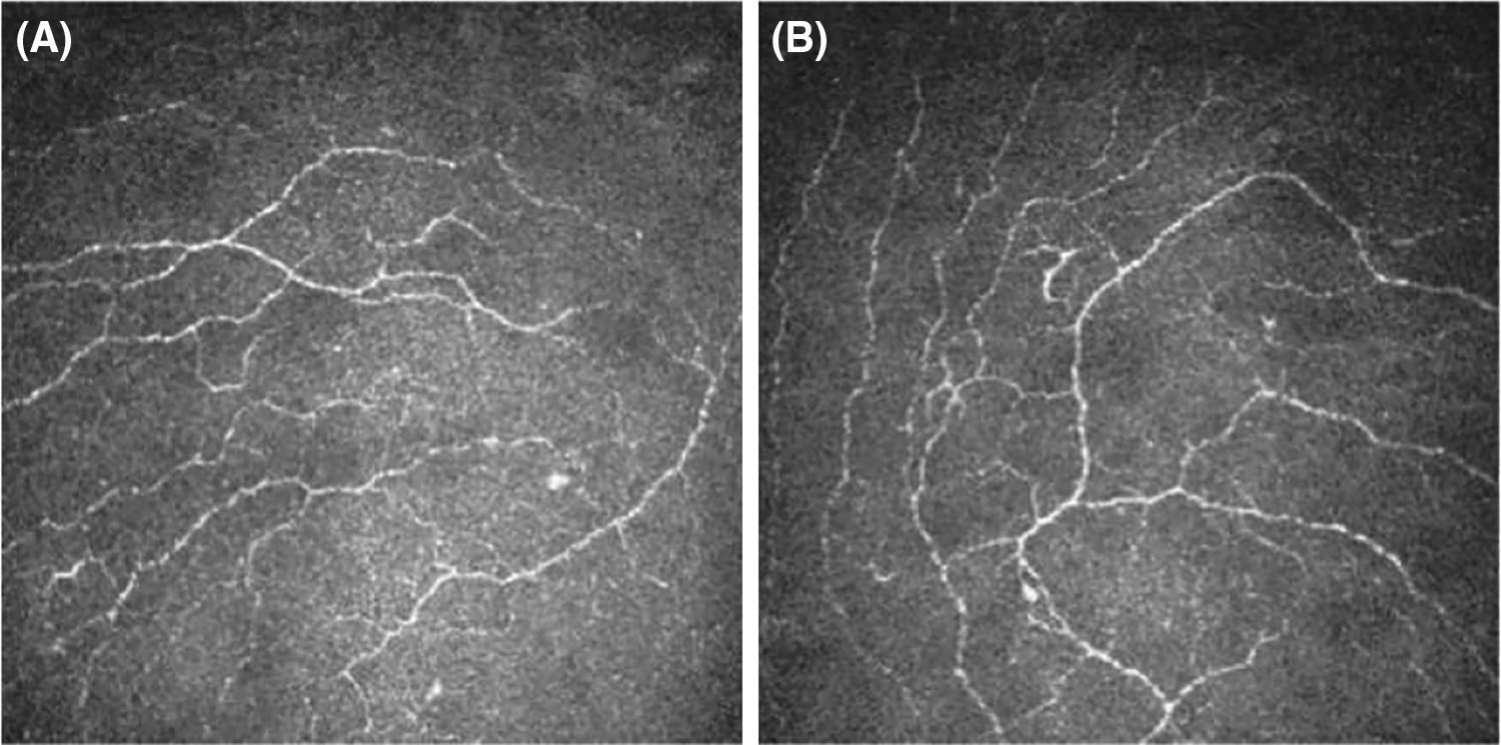
**The direction of nerve rotation in the corneal whorl-like area.** Note: (**A**) Clockwise and (**B**) counterclockwise.

A total of 60 cases and 120 eyes were included, and 20 cases and 40 eyes were in the control group, of which 20 cases and 40 eyes were in each group of the experimental group. There was no statistically significant difference (*P* > 0.05) between the groups in terms of gender and duration of disease; the difference between the groups in terms of glycosylated hemoglobin was statistically significant (*P* < 0.05; Table 1). After statistical analysis, the effect of age on corneal nerve parameters has been ruled out. The difference in the direction of nerve rotation in the corneal whorl-like area between the groups was not statistically significant (*P* = 0.402). Compared with the control group, the frequency of counterclockwise direction appeared increased in the diabetic group. It can be speculated that as DR progresses, the direction of nerve rotation in the corneal thread area changes (Fig. 2).

**Table 1.** Comparison of Basic Information of Four Groups

	Sexuality				
	Male	Female	Age, Y	Duration of Disease, Y	HbA1c (%)
Control (*n* = 40)	8 (18.2%)	12 (33.3%)	45.85 ± 11.72		4.8 ± 0.48
NDR (*n* = 40)	14 (31.8%)	6 (16.7%)	48.25 ± 12.2	9.25 ± 7.03	8.06 ± 2.12
mNPDR (*n* = 40)	12 (27.3%)	8 (22.2%)	56.4 ± 7.18	11.75 ± 5.39	8.71 ± 1.41
sNPDR (*n* = 40)	10 (22.7%)	10 (27.8%)	56.5 ± 7.81	14.05 ± 6.36	8.19 ± 1.39
X2/F	X2 = 4.04		F = 6.099	F = 2.909	F = 29.269
*P* value	0.257		0.001	0.063	*P* < 0.001

mNPDR, mild or moderate nonproliferative diabetic retinopathy; NDR, no diabetic retinopathy; sNPDR, severe nonproliferative diabetic retinopathy.

Quantitative variables are expressed as the mean value ± SD, and qualitative variables are expressed as the total number (percentage). Significance of the comparison was determined by chi-square test and 1-way ANOVA, and the LSD-*t* test was used for multiple comparisons between the two groups. The *P* values < 0.05 were considered significant.

The difference between RNFL values in the diabetic groups compared with the control group was statistically significant (*P* < 0.001), which showed that the mean value of RNFL thickness at the early stage of DR was reduced compared with the control group. Compared with the control group, the RNFL value of the patients with moderate NPDR and severe NPDR was statistically significant, that is, the RNFL value of patients with DR was significantly lower than that of patients without DR.

**Figure 3.**
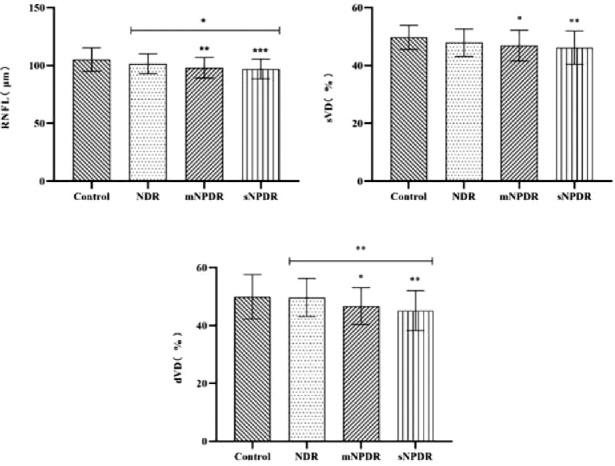
**Comparison of retinal nerve fiber layer (RNFL), superficial vascular density (sVD), and deep vascular density (dVD) in four groups.** Note: * Represents *P* < 0.05, ** represents *P* < 0.01, and *** represents *P* < 0.001.

When comparing the sVD and dVD among the four groups, it was found that the sVD and dVD in the moderate NPDR and severe NPDR groups were statistically significant compared with the control group (*P* = 0.012, 0.002, 0.041, and 0.002), and in the comparison between the four groups of dVD, the severe NPDR group was also statistically significant compared with the NDR group (*P* = 0.004), which showed that the mean values of sVD and dVD in early DR were reduced compared to the control group, and compared with patients without DR, the sVD and dVD of patients with DR decreased significantly (Fig. 3).

**Figure 4.**
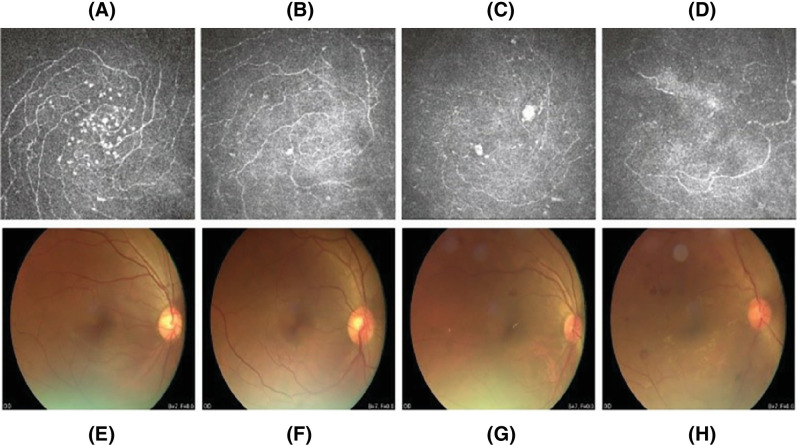
**Comparison of nerve in the whorl-like area of the cornea and retinal fundus image in four groups.** Note: (**A**) Nerves in the corneal whorl-like area in the control group. (**B**) Nerves in the corneal whorl-like area in the NDR group. (**C**) Nerves in the corneal whorl-like area in the moderate NPDR group, (**D**) Nerves in the corneal whorl-like area in the severe NPDR group. (**E**) Retinal fundus image in the control group. (**F**) Retinal fundus image in the NDR group. (**G**) Retinal fundus image in the mild to moderate NPDR group. (**H**) Retinal fundus image in the severe NPDR group.

The differences were statistically significant when comparing IWL, CNFL, and FLV among the four groups (*P* < 0.001), which showed that as the degree of diabetic retinopathy increased in the diabetic group, the mean value of IWL and CNFL decreased compared with the control group, and FLV increased compared with the control group (Fig. 4). It can be considered that the IWL is related to the severity of DR. At the same time, compared with patients without DR, the IWL of patients with DR decreased significantly. There was no significant difference in GLV between the groups (*P* = 0.3), but GLV tended to increase in the diabetic group compared with the control group. Compared with the control group, the GCC in the diabetic group tended to decrease, but the difference was not statistically significant (*P* > 0.05; Table 2).

**Table 2.** Comparison of Ophthalmic Parameters Among Four Groups

*n* = 40	Control	NDR	mNPDR	sNPDR	F/H	*P* Value
RNFL (µm)	105.1 ± 10.12	101.4 ± 8.6	98.03 ± 8.97^*^	96.88 ± 8.52^*^^,^^†^	F = 6.658	P < 0.001
sVD (%)	49.72 ± 4.17	47.89 ± 4.76	46.85 ± 5.31^*^	46.13 ± 5.73^*^	F = 3.829	0.011
dVD (%)	49.91 ± 7.65	49.71 ± 6.59	46.74 ± 6.34^*^	45.15 ± 6.89^*^^,^^†^	F = 4.569	0.004
IWL (mm/mm²)	26.34 (25.88, 27.55)	22.41 (22.01, 22.91)^*^	18.66 (18.45, 18.97)^*^^,^^†^	17.7 (16.52, 18.65)^*^^,^^†^^,^^‡^	H = 139.178	*P* < 0.001
CNFL (mm/mm²)	23.27 (22.61, 23.73)	21.58 (21.02, 22.01)^*^	18.03 (17.86, 18.54)^*^^,^^†^	16.18 (15.99, 16.52)^*^^,^^†^^,^^‡^	H = 144.874	*P* value < 0.001
GCC (µm)	102.5 (98, 108)	100 (98.25, 104)	100 (96.25, 102)	94.5 (91, 102.75)^*^^,^^†^	H = 13.936	0.003
GLV (%)	0.56 (0.18, 1.64)	0.88 (0.47, 2.05)	1.16 (0.47, 1.85)	0.59 (0.11, 2.8)	H = 3.664	0.3
FLV (%)	0.31 (0.09, 0.6)	0.85 (0.48, 1.83)^*^	2.63 (1.09, 3.52)^*^^,^^†^	6.2 (4.45, 7.68)^*^^,^^†^^,^^‡^	H = 74.648	*P* value < 0.001

CNFL, central nerve fiber length; dVD, deep vascular density; FLV, focal loss volume, GCC, ganglion cell complex; GLV, global loss volume; IWL, inferior whorl length; RNFL, retinal nerve fiber layer; sVD, superficial vascular density.

The measurement data obeyed normal distribution and were expressed as mean ± standard deviation, and 1-way ANOVA was used for comparison among three groups, and LSD-*t* test was used for multiple comparisons between two groups. The non-normal distribution was expressed as median (lower quartile, upper quartile; M [P25, P75]). The Kruskal-Wallis H test was used for comparison among the three groups, and the Bonferroni (post hoc test) method was used to correct for multiple comparisons between the two groups. The *P* values < 0.05 were considered significant.

* Represents significant differences compared to the control group.

† Represents significant differences compared to the NDR group.

‡ Represents significant differences compared to the moderate NPDR group.

**Figure 5.**
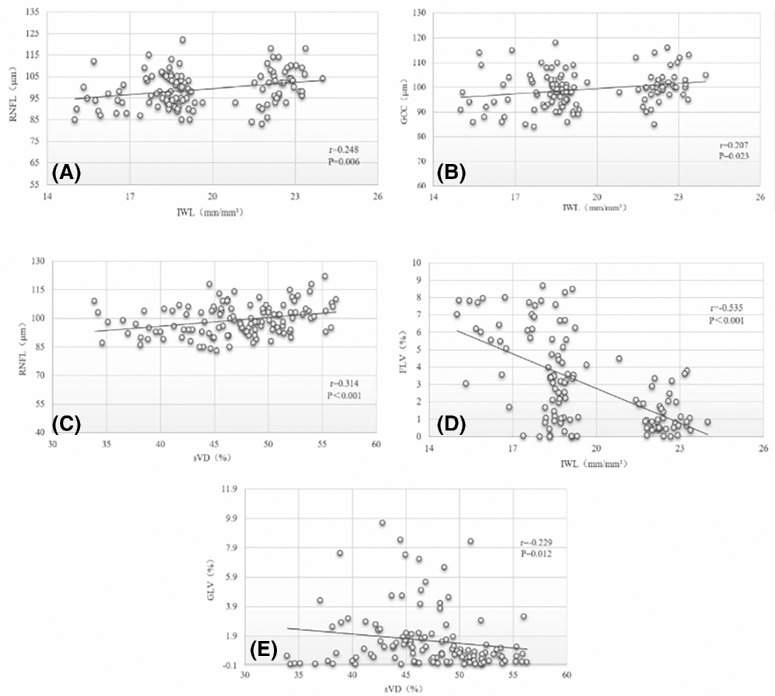
**Correlation analysis in four groups.** Note: (**A**) Correlation between IWL and RNFL. (**B**) Correlation between IWL and GCC. (**C**) Correlation between sVD and RNFL. (**D**) Correlation between IWL and FLV. (**E**) Correlation between sVD and GLV. RNFL, retinal nerve fiber layer; sVD, superficial vascular density; dVD, deep vascular density; IWL, inferior whorl length; GCC, ganglion cell complex; GLV, global loss volume; FLV, focal loss volume.

Correlation analysis of nerve length in the corneal whorl-like area with retinal nerve and retinal microcirculation parameters was performed. The results showed that IWL in early DR was significantly and positively correlated with RNFL, GCC, and dVD (*r* = 0.248, *P* = 0.006; *r* = 0.207, *P* = 0.023; and *r* = 0.234, *P* = 0.01, respectively), and IWL was significantly and negatively correlated with FLV (*r* = −0.535, *P* < 0.001; Table 3).The results also showed that sVD was significantly and positively correlated with RNFL and dVD in early DR (*r* = 0.314, *P* < 0.001 and *r* = 0.343, *P* < 0.001, respectively), and sVD was significantly and negatively correlated with GLV (*r* = −0.229, *P* = 0.012). Correlation analysis was performed between deep vessel density and other parameters. The results showed that dVD in early DR was significantly and positively correlated with IWL and sVD (*r* = 0.234, *P* = 0.01 and *r* = 0.343, *P* < 0.001, respectively; Fig. 5).

**Table 3.** Correlation Analysis Between IWL and Each Index

	r	*P* Value	*n*
sVD (%)	0.159	0.083	120
dVD (%)	0.234^*^	0.01	120
RNFL (µm)	0.248^**^	0.006	120
GCC (µm)	0.207^*^	0.023	120
GLV (%)	−0.094	0.309	120
FLV (%)	−0.535^***^	P < 0.001	120

dVD, deep vascular density; FLV, focal loss volume; GCC, ganglion cell complex; GLV, global loss volume; IWL, inferior whorl length; RNFL, retinal nerve fiber layer; sVD, superficial vascular density.

Spearman rank correlation analysis was used for the correlation analysis among the factors. The *P* values < 0.05 were considered significant.

* Represents *P* < 0.05.

** Represents *P* < 0.01.

*** Represents *P* < 0.001.

## Discussion

DPN is the most common diabetic neuropathy and has a high incidence among people with diabetes. At present, the intra-epidermal nerve fiber density (IENFD) test is considered the gold standard for diagnosing DPN, but this method for detecting small fiber lesions is invasive and has limited clinical application, so a more accurate and acceptable test is currently needed. The cornea is one of the most densely distributed nerve tissues in the body and consists of small nerve fibers of the thin medullary Aδ and unmyelinated C classes, which can be involved in the early stages of DPN. Several studies have confirmed that diabetic corneal neuropathy (DCN) is the first evidence of subclinical DPN and progresses together with the course of the disease,^9^^,^^18^ and examinations of the corneal nerve can yield sensitive markers for the early diagnosis of DPN.

The corneal nerve emerges from the trigeminal nerve, and the nerve bundle passes vertically through the anterior elastic layer and then walks flatly on the surface of the cornea to form the sub-basal plexus. The sub-basal plexus of the cornea, most widely studied site in DR, is distributed between the basal cells of the corneal epithelium and the anterior elastic lamina, forming a whorl-like structure approximately 1 to 2 mm nasal to the inferior corneal apex.^10^ The whorl-like area is an ideal anatomic landmark for observing corneal nerve changes because of its fixed location, ease of identification, and reproducibility with respect to the central corneal area.^10^^,^^11^ In addition, Badian et al. found that in patients with diabetes and Parkinson's disease, these structures are oriented in a more counterclockwise direction, and the bilateral symmetry is lost,^10^ which was consistent with the conclusions of us. But whether this index can be used as diagnostic indicators of neurodegenerative diseases requires further examination. In the present study, the IWL was significantly smaller in patients with diabetes than in controls and progressively shrank with the progression of diabetes, consistent with the findings of Ioannis et al.^14^

There are several theories about the mechanism of formation of corneal nerve threads; the earliest studies suggested that it is related to the migration and growth of corneal epithelial cells, some scholars have suggested its association with chemical guidance and electromagnetic indexing.^19^ Furthermore, other studies have found that the growth of the corneal epithelium and corneal basal plexus nerves in mice are not synchronized,^20^ so this theory remains controversial. The human corneal basal plexus whorl-like structure coincides with the Fibonacci spiral, which is usually found in fast-growing plants,^21^ and a similar spiral structure has been found in the human scalp. The helical structure of the scalp is thought to be determined by the tension on the epidermis as the hair follicle grows downward during rapid cranial expansion.^22^ During human blinking, the upper and lower eyelids meet approximately 2 to 3 mm below the horizontal meridian, that is, at the level of the threaded area,^23^^,^^24^ and patients with keratoconus and those wearing orthokeratology lenses also exhibit changes in the structure of corneal nerve threads. Therefore, the pressure and mechanical forces generated by the rapid growth of the eyeball and mechanical action may have an effect on the formation of neural patterns in threaded structures. Diabetic peripheral neuropathy is a nerve fiber length-dependent distal neuropathy,^25^ and, in these patients, corneal nerve fiber damage shows a progressive trend from the central to the whorl-like area, and changes in the nerves in the corneal whorl-like area precede those in the central corneal area and correlate with disease progression.^12^^,^^15^ In our research, we evaluated CNFL and IWL to assess the proximo-distal effect on corneal nerves. We also found that the length of the nerve in the whorl-like area is higher than that in the central area, and as the nerve moves from the central area to the whorl-like area, the length of the nerve gradually decreases between different groups, so it is consistent with the existing mechanism of diabetic peripheral neuropathy, as the corneal nerve whorl-like zone is the most terminal part of the corneal sub-basal plexus.

DPN and DR are common complications of diabetes, and there is a known association between their severities. Current studies have found that retinal neurodegeneration may occur earlier than vascular lesion formation in the pathological process of DR.^26^ The RNFL is associated with a reduction in the mean thickness of the ganglion cell layer (GCL) and inner plexiform layer (IPL) at a rate similar to that of patients with severe glaucoma.^26^ Among the cells of the retina, the retinal ganglion cells (RGCs) are lost the earliest and demonstrate the highest rate of apoptosis. The RNFL represents the axons of the RGCs, and the GCL and IPL represent their cell bodies and dendrites; therefore, changes in these layers can well reflect the changes that occur in the RGCs.

In our study, we found that the RNFL thickness in the case group was significantly lower than that in the control group and gradually decreased in both groups as the degree of DR increased, which is consistent with the results of Jia et al.^4^ and Hafner et al.^26^ In addition, we found that compared with that in the control group, patients with nonproliferative DR had begun experiencing a reduction in the GCC thickness and RGC loss relative to the control group, which is consistent with the conclusions of Frizziero et al.^3^ In addition, we found that the FLV was significantly higher in the case group than in the control group, and that the FLV gradually increased between groups as DR worsened, consistent with the conclusions of Srinivasan et al.^27^ This indicates that the percentage of individuals with locally significant RGC losses increases as DR progresses. The results showed that the local damage to the GCC was significant and could be reflected in the FLV in patients with DR as the disease progresses, acting as one of the best indicators for the early identification and severity of DR. The GLV in the NDR group and moderate NPDR group was higher than that in the control group, but the difference was not significant, consistent with the study of Srinivasan et al.^27^ However, unlike the latter study, there was no significant change in the GLV of severe NPDR group with respect to the control group in this experiment. Additionally, Bontzos et al.^28^ found no significant difference in the GLV value between the NDR group and the control group. It is speculated that the GLV is less sensitive to early DR and may require more widespread and diffuse RGC loss to affect its value, whereas extensive RGC loss mostly occurs in late DR. Therefore, the role of GLV in early DR injury needs to be verified.

The insidiousness of the early symptoms of DR and the inability to determine the degree of DR in some patients through fundus examination hinder the early clinical diagnosis and intervention of DR. Therefore, we discussed DPN and DR to explore the connection between the corneal cord nerves and retinal nerves in the early stage of DR. Previous studies have shown that RNFL changes are significantly associated with DPN,^26^ and the FLV of the GCC has been suggested as an independent predictor of the occurrence of DPN. IVCM detection of corneal nerve-related indicators is the gold standard for DPN,^18^ and because the corneal nerve and the retinal nerve are part of the central nervous system, it is hypothesized that there may be a close correlation between the two. Hafner et al.^29^ concluded that the degree of DPN is highly correlated with that of DR according to their analysis of the central corneal nerve and retinal nerve in patients with diabetes, which is consistent with the results of this study. Here, the IWL in the case group significantly decreased as the RNFL and GCC thicknesses decreased and the FLV increased. Unlike previous experiments, the present experiment was the first to analyze the IWL and retinal nerves in diabetic patients. Therefore, we hypothesized that the corneal IWL in patients with diabetes could be used as one of the observational indicators of DR for early identification before the onset of clinically detectable DR and to prevent serious complications.

The relationship between neurodegenerative changes and blood flow changes in DR remains controversial. The blood vessels and nerves in the retina, named neurovascular units (NVUs), demonstrate a notable homeostasis, and when hyperglycemia in the body leads to cellular dysfunction, structural changes and dysfunction of the NVUs occur, including loss of neurovascular associations, neurodegeneration, glial cell proliferation, and neuroinflammation,^30^ leading to further cellular damage, all of which occur before vessel alterations become clinically observable. In this study, we showed that the sVD and dVD were significantly lower in the case group than in the control group, and as the degree of DR increased, the sVD and dVD values gradually decreased between the groups. This suggests that retinal perfusion is significantly reduced in the early stages of DR and that microvessel damage has already occurred, consistent with the studies of Kim et al.^6^ and Hafner et al.^26^ In this experiment, the FLV was significantly elevated in the NDR group, whereas the sVD and dVD were not significantly changed, from which we speculate that the onset of neurodegenerative changes may precede blood flow changes in DR. Li et al.^31^ found significantly lower blood flow density in patients with NDR, possibly due to differences in sample sizes or between individuals.

At present, some scholars believe that DR first leads to apoptosis of retinal nerve cells and damages the blood-retinal barrier, and then develops into microangiopathy.^32^ In this experiment, we demonstrated that there is an inextricable relationship between the retinal nerves and blood vessels during DR. There were significant changes in the IWL and FLV in the NDR group relative to the control group, so we speculate that in early diabetic retinal lesions neuropathy may occur first before vessel lesions, which then affects the neurovessel unit and induces vessel changes. However, this does not mean that neuropathy necessarily precedes vessel lesions because the detection means may be limited, preventing the ability to detect early microvessel changes, so the connection between nerves and blood vessels in the natural course of DR and the specific mechanisms need further study in the future.

## Conclusions

In conclusion, the experimental results showed that the IWL decreases significantly with the progression of DR, and a correlation between the corneal whorl-like nerves and retinal nerves in patients with early DR was identified, which suggests that the changes in the nerves in the corneal whorl-like area may be an early sign of DR. In some patients with diabetes who are unable to cooperate with an OCTA examination due to refractive interstitial clouding, such as cataract or vitreous opacity, we can estimate the presence of retinopathy by corneal confocal examination. Any conclusions regarding the exact temporal relationship between neuropathy and vessel disease need to be further explored in larger longitudinal studies, and we will continue to conduct longitudinal studies with long-term follow-up of patients in the future to form a series of studies on DR.
